# Catastrophizing Has a Better Prediction for TMD Than Other Psychometric and Experimental Pain Variables

**DOI:** 10.1155/2020/7893023

**Published:** 2020-11-12

**Authors:** Lisa Willassen, Anders Arne Johansson, Siv Kvinnsland, Kordian Staniszewski, Trond Berge, Annika Rosén

**Affiliations:** ^1^Department of Clinical Dentistry, University of Bergen, Bergen, Norway; ^2^Department of Oral and Maxillofacial Surgery, Haukeland University Hospital, Bergen, Norway

## Abstract

Temporomandibular disorders (TMDs) are characterized by moderate to severe pain in the masticatory muscles and/or the temporomandibular joint (TMJ). The present study is a part of a multidisciplinary project, initiated by the Norwegian Ministry of Health. The main purpose of this study is to compare a cohort of TMD patients to healthy individuals regarding experimental pain, the degree of disability caused by living with pain and psychometric variables, and to investigate which of these variables is the best predictor for TMD patients. We hypothesised that TMD patients have more disability when living with pain and lower pain thresholds than healthy controls, and those psychometric variables are stronger predictors than pain thresholds provoked by experimental pain. Sixty TMD patients were matched by sex and age to sixty healthy individuals without TMD symptoms or other musculoskeletal symptoms in the head and neck region. All subjects completed a questionnaire that included psychometric characteristics, that is, a one- and two-item version of the Pain Catastrophizing Scale, the Hospital Anxiety and Depression Scale (HADS), and the Roland Morris Scale (RMS), which measures disability when living with pain. They also underwent a clinical examination including the measurement of pain thresholds with electrical and pressure stimuli. The TMD patients had lower pain thresholds for experimental electrical and pressure stimuli compared with the controls (*P* < 0.05 and <0.001, respectively). They also scored higher than healthy individuals with disability living with pain (*P* < 0.001), anxiety (*P* < 0.001), depression (*P* < 0.001), and catastrophizing (*P* < 0.001). The results for anxiety, depression, and catastrophizing have been published earlier, and the reused data in this study are compared with RMS and pain thresholds. The conditional logistic regression model identified catastrophizing (OR = 2.42, CI 1.22–4.79) as a significant predictor of TMD patients. The results support this hypothesis and indicate that TMD patients have lower pain thresholds and more disability when living with pain compared to healthy individuals, where the strongest prediction for TMD was catastrophizing. Awareness of psychometric disabilities in TMD patients is of importance when considering the choice of treatment.

## 1. Introduction

The conditions that cause pain and/or dysfunction of the temporomandibular joint (TMJ) and muscles that regulate jaw movements are collectively known as temporomandibular disorder (TMD). In a TMD population, the disorder is assumed to be at least three times as common in women as in men [1], while in the general population, TMD is assumed to be two times as common [2]. The overall prevalence in the general population is around 3% to 12% among 30- to 50-year-old individuals [3, 4]. According to a prospective study of adults in the United States, the annual incidence of TMD onset is 4% [5]. TMD-related pain usually occurs periodically over time and can be mild, moderate, or severe. In most cases of TMD, symptoms can usually be managed with simple reversible conservative treatment [6]. However, some patients with painful TMD experience prolonged chronic pain and reduced function of the masticatory system, both of which can be treatment-resistant. Prolonged and intense TMD symptoms may have severe consequences for patients, including psychological, physical, behavioural, and psychosocial problems [1]. Patients with TMD often have impaired mandibular function, with difficulties in jaw movement such as chewing, yawning, speaking, and even kissing as the dominant problems. Comorbidities often occur, particularly general pain conditions, such as other systemic joint diseases, headaches, and ear and eye pain [1, 7, 8].

Understanding the source of pain is important for making a diagnosis and for choosing the appropriate treatment, which may be conservative, psychological, and/or surgical [9]. The pathophysiology of TMD is currently unknown, but pain amplification (abnormal pain sensitivity), central sensitisation, and changes in immune activity have been associated with TMD [10]. A prospective genetic-based study found that individuals who are more sensitive to pain have a significantly higher probability of developing painful TMD than patients who are less sensitive [11].

Sensitivity of tissues can be assessed by measuring pressure pain thresholds (PPTs) with algometry. It is a valid and reliable method used to measure the PPT in craniocervical muscles [12]. In tension-type headaches, decreased pressure pain thresholds over the craniocervical area have shown to reflect signs of the sensitisation of the trigeminocervical nucleus caudalis [13]. In the orofacial area, experimental pain, induced by pressure [14–16] or electricity [17], determines individual pain thresholds or objective pain. Pressure is used to activate mechanosensitive receptors and quantify deep muscle pain. Electricity activates non-nociceptive and nociceptive afferents, affecting tissue that is more superficial [18]. An electrical stimulus can gradually be increased, and subjects have to report when the stimulus changes from a feeling of sensation to a feeling of pain. This neurophysiological model of sensitisation of the trigeminocervical nucleus caudalis is generally presumed to play an important role in the onset and maintenance of migraine and chronic tension-type headaches [19]. Chronic headaches are common in TMD, a well-known comorbidity [7]. Previous studies have shown low pain thresholds in TMD patients in response to noxious stimuli [20–22].

Questionnaires are often used to assess self-reported symptoms such as pain (subjective pain) and psychometric status. Psychosocial factors are suggested as linked to pain-related disability and duration of pain [23]. Pain is found to be strongly associated with specific anxiety and depressive disorders [24], and the presence of anxiety and depression is found to be associated with higher muscle tenderness in patients with different types of facial pain [25]. High scores for anxiety/depression and pain catastrophizing are commonly reported in TMD patients [26–28]. Comorbid anxiety and depressive disorders are associated with disability, impairment, decreased quality of life, increased health care utilisations, and substance use in individuals with pain disorders and symptoms [24].

Patients with TMD, who will undergo TMJ surgery, may have a high risk of ultimately experiencing postoperative pain. Among the patients included in this study, seven patients underwent TMJ surgery, and only one of them was pain-free at the follow-up [29]. According to the guidelines from the International Association for the Study of Pain (IASP), patients with psychological disorders should receive treatment for such disorders prior to surgery to prevent the development of persistent postoperative pain. Such pain is more likely when preoperative pain, fear of pain, expected pain, and catastrophizing are present [30]. An earlier study assessed if preoperative psychological testing could predict the outcome after arthroscopy, and a weak statistically insignificant association was found between chronic anxiety and pain in TMD patients after surgery. However, the authors addressed the need for further studies in order to clarify the role of chronic anxiety for the outcome of TMJ surgery [31]. Currently, there exist several studies suggesting the relationship between experimental pain, psychometric variables, and TMD [5, 32, 33], but to our knowledge, few studies explore the combined significance of experimental pain thresholds/psychometric variables regarding TMD. An earlier study on the same cohort as presented in this study found higher scorings for anxiety, depression, and catastrophizing in TMD patients compared to healthy controls [34]. These psychometric data are reused in the present study in order to perform inferential analyses with new findings and execute more advanced statistical analysis.

The main purpose of this study is to compare a cohort of TMD patients to healthy controls regarding experimental pain, disability when living with pain and psychometric variables, and to investigate which of these variables was the best predictor for TMD patients. We hypothesised that TMD patients have more disability when living with pain and lower pain thresholds than healthy subjects. We further hypothesised that psychometric variables are stronger predictors for TMD than pain thresholds provoked by experimental pain.

## 2. Materials and Methods

Under direction from the Norwegian Directorate of Health, the Oral and Maxillofacial Surgery Department and the Clinic for Pain Treatment and Palliation at Haukeland University Hospital in Bergen, Norway, developed a multidisciplinary investigation programme for patients with severe TMD [9].

### 2.1. Participants

The participants in this study consisted of sixty consecutively referred patients with severe TMD and sixty age- and gender-matched healthy controls. The study groups to be characterized were set at sixty patients by the directive from the health directorate. The sixty TMD patients included in the study were referred from their general medical practitioner during 2013 to 2015 for severe TMD with long-lasting pain. The consecutively included patients were assessed for TMD with a modified DC/TMD, without using the mandatory command, which has previously been shown not to impair diagnostic reliability [35]. Inclusion criteria for admission to the programme included TMD-related pain in the orofacial area, decreased function of the jaw, and general disability because of pain. Exclusion criteria included current substance abuse or severe psychiatric diagnoses. Subjective symptoms and clinical signs were assessed by a multidisciplinary team consisting of specialists in oral and maxillofacial surgery, specialists in orofacial pain, a pain physician, a psychologist, a physiotherapist, and a radiologist. The mean duration of pain for the patients was 11 years. TMD main diagnoses comprised myalgia (*n* = 22), arthralgia (*n* = 1), disc derangement (*n* = 2), and combinations thereof (*n* = 35).

Sixty healthy age- and sex-matched subjects without symptoms of TMD were recruited to serve as a control group. The inclusion criteria for the controls were that they were age- and gender-matched to each of the participants in the patient group. The exclusion criteria included TMD symptoms and pain symptoms in the head and neck. The participants in the control group were a convenience sample selected from the Department of Clinical Dentistry at the University in Bergen. All included patients and controls signed a consent form for participating in the study before the investigations. Two different examiners assessed the groups, one for the control group and one for the TMD group. Both examiners underwent specific correlation/synchronisation training before the clinical assessments.

Recorded details for all patients were stored in their hospital medical records (DIPS). Data were collected in an anonymised form. The project was approved by the Regional Committee for Medical and Health Professional Research Ethics (2015/930/REK sør-øst).

### 2.2. Measurements

#### 2.2.1. Subjective Self-Reported Measurement

All study participants completed a questionnaire, the Roland Morris Scale (RMS), and an additional questionnaire assessing general disability when living with pain [36]. RMS consists of 24 questions/claims as a measure of disability when living with chronic pain. The participants marked claims that were correct with an *X* (1 point for each claim; maximum score = 24 points and cut-off = 7 points).

The psychometric data from the already published study [34] included the two-item version of Coping Strategies Questionnaire (CSQ) regarding catastrophizing [37] and the Hospital Anxiety and Depression scale (HADS) [38]. The CSQ included two questions which ranged from 0 to 6 points, where 0 was the lowest score and 6 the highest score (a total of 6 + 6 points, cut-off >1 for each question). The HADS included 7 questions regarding anxiety and 7 questions regarding depression. Each question could be answered in 4 different ways, ranging from 0 to 3 points (a total of 21 points and cut-off ≥8 for each condition).

#### 2.2.2. Experimental Pain: Assessment of Sensitivity and Pain Thresholds

To assess pain sensitivity and hyperalgesia, pressure and electrical stimuli were used. An algometer assessed pressure pain thresholds (PPTs) on the TMJ, the masseter muscle, and the finger [39, 40]. The algometer (Somedic, Hörby, Sweden) had a probe with a surface area of 1.0 cm^2^ and a slope of 30 kPa/s. The algometer was equipped with a warning signal to prevent overload and a green light to indicate correct pressure. The assessment was performed on the TMJ and the most prominent part of the masseter muscle, representing local pain. The patients were asked to occlude their teeth and then relax their jaw to enable the examiner to find the most prominent part of the masseter muscle prior to the algometer measurement. Algometry was also performed on the tip of the pointing finger, representing global pain. The examiner placed the probe on the area being tested, and as soon as the subject perceived pain (PPT), they pushed a button to register the exact value of the weight used. A computer registered and displayed the pressure value. All tests were performed three times bilaterally, and the mean of the measured values was recorded.

The electrical stimuli pain test was performed on the fingers, as for the algometer, representing global pain. We included two measurements, specifically electrical sensibility thresholds (ESTs) and electrical pain thresholds (EPTs), using the PainMatcher (PainMatcher AB, Lund, Sweden). The PainMatcher is a microprocessor, which delivers a constant current of 15 mA with monophasic pulses of 10 Hz to the electrodes. Finger press on the electrode ensures an electrically closed circuit and increased intensity of the pulse, which is sustained for 4 *μ*s to a maximum of 396 *μ*s [39]. In the first test, subjects were asked to release the electrode as soon as the stimulus was felt. In the second test, subjects were asked to release the electrode as soon as they felt the first feeling of pain from the stimulus. A number corresponding to the intensity of the electric stimulus was displayed on the apparatus (score 0–99). The tests were repeated three times, and the mean value was calculated.

### 2.3. Statistical Analyses

Descriptive data were analysed, and the Wilcoxon signed-rank test was used for bivariate analyses between TMD cases and controls. The results from HADS and catastrophizing have been published earlier as single independent variables, but the present results are novel with respect to the adjusted conditional logistic regression [34]. Multivariate conditional logistic regression using an unadjusted Wald test, and an adjusted one with a stepwise forward procedure including five variables (PPT in the masseter muscle/finger and scores from catastrophizing, depression, and anxiety scales), was performed. Selection criteria for the independent experimental pain and psychometric variables were of theoretical relevance among the variables showing significant associations in bivariate comparisons between TMD cases and controls (Wilcoxon signed-rank test).

The data analysis was performed in IBM SPSS Statistics for Macintosh, version 25.0 (IBM Corp., Armonk, N.Y., USA), and Stata Statistical Software: release 14, College Station, TX: StataCorp LLC. *P* < 0.05 was considered a statistically significant difference.

## 3. Results

### 3.1. Descriptive Data

#### 3.1.1. Study Population

**Figure 1:**
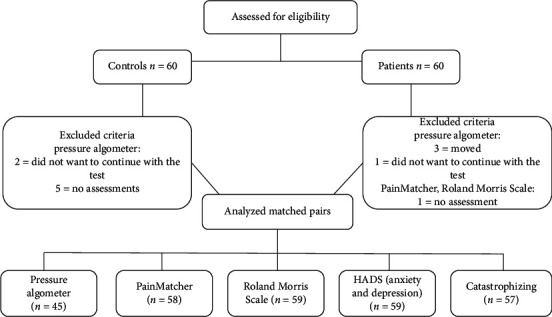
Flowchart of the study sample.

The TMD group had a mean age of 45 years (SD 12.6) and included 51 women and 9 men. The mean age of the age- and sex-matched control group was 46 years (SD 12.6), and it consisted of 51 women and 9 men. Because some patients dropped out or moved during the study, we ultimately had a smaller number of participants and matched pairs (Figure 1): pressure algometer (*n* = 45), PainMatcher (*n* = 58), Roland Morris Scale (*n* = 59), HADS (*n* = 59), and catastrophizing (*n* = 57).

### 3.2. Experimental: Assessment of Sensitivity and Pain Thresholds

The results show that the TMD patients had a lower PPT than the controls (*P* = 0.001 for the finger and masseter muscle and *P* = 0.003 for the TMJ).

Results from the analysis of the EPT measurements indicate significant differences between the two groups, with the TMD patients scoring lower than the controls (*P* = 0.014). However, the EST measurements did not differ significantly between the TMD group and the controls (Table 1).

### 3.3. Self-Reported Measurements

The results from the RMS showed increased disability for TMD patients compared to controls (*P* = 0.001, positive score *n* = 33 for the patients and *n* = 2 for the controls; cut-off *P* < 0.001). Furthermore, the TMD patients had more anxiety (*P* = 0.001, positive score *n* = 22 for the patients and *n* = 5 for the controls; cut-off *P* < 0.001), depression (*P* = 0.001, positive score *n* = 16 for the patients and *n* = 1 for the controls; cut-off *P* < 0.001) and catastrophizing (*P* = 0.001, positive score *n* = 52 for the patients and *n* = 11 for the controls; cut-off *P* < 0.001) compared to the controls. Descriptive data are shown in Table 1. The results regarding anxiety, depression, and catastrophizing have previously been published [34]. In this study, those results were used to perform inferential analyses with data from the RMS, HADS, and experimental pain in order to execute more appropriate statistical analyses, i.e., conditional regression.

### 3.4. Conditional Logistic Regression Analysis

Multivariate conditional logistic regression using an unadjusted Wald test and an adjusted model using stepwise forward procedure included five independent variables: PPT in the masseter muscle and finger as well as scores from catastrophizing, depression, and anxiety scales. Unadjusted and adjusted models are shown in Table 2. Adjusted conditional logistic regression identified catastrophizing (OR = 2.42, CI 1.22–4.79, Table 2) to be the only significant predictor. The Nagelkerke was 0.917. The results from the Wald test and stepwise forward test regarding catastrophizing did not appear identical. This can be explained by a reduced number of matched pairs in the adjusted analysis due to missing values in the included independent variables.

## 4. Discussion

Decreased PPT and higher scorings for psychological factors in TMD patients compared to healthy controls are well known in the literature [5, 14, 15, 21, 27, 32, 41, 42], but the relationship of different factors with predicting TMD patients is yet to be described. To our knowledge, this study is the first to analyse the interrelationship between experimental pain thresholds and psychometric variables in relation to TMD by using a multivariate regression model. Thus, our study demonstrates that the catastrophizing has the best prediction of TMD compared to other psychometric variables (anxiety and depression) and experimental pain thresholds (EPT and PPT). In this regard, a previous study found that high levels of pain catastrophizing increased the risk of pain and disability in chronic back pain patients [43]. A study by Sorbi et al. using electronic EMS diaries suggests that both pain intensity and psychological variables explain disability in chronic pain disorders (CPDs), as well as substantiate the relevance of psychological functioning for disability in CPD. They found that the prediction of disability by avoidance behaviour, pain-related fear, and catastrophizing was better compared to pain intensity and stronger in pain of longer duration than pain of shorter duration [44]. Another study found that high-pain catastrophizing TMD patients were similar to patients with other chronic pain conditions and supported the decision to add scoring for pain catastrophizing to the DC/TMD in order to identify TMD patients who are at the risk of developing chronic pain [41]. The present study supports the aforementioned reports and indicates that catastrophizing might be of causal importance in the development and persistence of pain related to TMD. Moreover, it highlights the importance of assessing catastrophizing in the diagnosis and addressing it in the treatment of TMD.

In addition to the interesting findings in the multivariable analysis, the bivariate analyses showed significant differences between the TMD group and controls regarding not only catastrophizing but also anxiety, depression, and disability when living with pain and experimental sensitivity/pain thresholds. In this regard, it has previously been suggested that psychological factors are associated with the development of painful TMD [21], and enhanced pain sensitivity for experimental pain has been registered in TMD patients [20]. The OPPERA study recently published a community-based cohort study regarding risk factors and enduring characteristics in TMD patients. They studied risk factors using questionnaires and clinical measures, which included clinical, health, psychological, behavioural, and neurosensory domains. Risk factors from the psychological domain were, among others, anxiety, depression, and catastrophizing. The results indicate that nearly all risk factors from all domains increased in patients who developed TMD, while remaining in patients with persistent TMD and declining in those with transient TMD. This suggests that TMD pain onset is determined by enduring characteristics and changes in biopsychosocial functioning across time [45]. These results corroborate our study, which found high psychological scorings in patients with severe TMD and long-lasting pain. All of these findings could indicate that the intensity and duration of TMD are related to the severity and type of psychological impact and that fluctuations of TMD symptoms are dependent on psychological mechanisms. To further evaluate this speculation, future studies should have a longitudinal design, including a higher number of patients with different severity and duration of pain and robust measures of psychological impacts in order to evaluate its impact on painful TMD.

Previously published data, in the same cohort of patients as presented in this study, show higher scoring on HADS and CSQ compared to healthy individuals, as well as higher levels of cortisol in the saliva, showing an upregulated HPA axis indicating higher levels of stress [34]. These findings are also supported by others who have found that stress is a strong predictor of TMD pain [46]. Canales et al. found high to moderate levels of depression and somatisation in patients with TMD [47], and it has been established that concurrent depression and pain have a greater impact on chronic pain disorder than pain disorders alone [42]. Greater pain intensity, longer duration of pain, persistent pain, impaired social functioning, and the likelihood of poor treatment outcome are seen when depression and pain coexist [48]. A similar study assessing the potential role of biological, psychological, and social factors in order to predict the presence of painful TMD using multivariate analysis found a relationship between TMD pain and depression, which supports the need of considering both psychological factors in relation to TMD signs and symptoms [27].

An association between psychological distress, a lower threshold in experimentally induced pain, and painful TMD has been found [5] in analogy with the present study.

Not unexpectedly, the TMD patients in the present study had more tenderness measured by the PPT, and previous studies have reported greater sensitivity to experimental pain because of alterations in endocrine, sensory, and psychological processes, as well as central sensitisation [49, 50]. In tension-type headaches and migraines, decreased PPT in the trapezius muscle and suboccipital sites reflect altered pain perception and support the pathophysiological model of sensitisation [51]. On the contrary, Stuginski-Barbosa et al. [33] found a statistically weak correlation between pain intensity (shown on a visual analogue scale) and PPT (measured by an algometer) in TMD patients with arthralgia, suggesting that other factors, such as nociceptive processes in the central nervous system and additional psychological factors, are important in explaining pain in TMD patients. The difference in PPT between the TMD patients and controls in our study might have an association with the high psychological impact in the TMD group.

The surgically treated patients in this cohort, published in 2017, had high scoring of catastrophizing and postoperative pain [29], possibly due to surgical fear, expected pain, and preoperative pain [30, 52]. The suggestion that catastrophizing might be a predictive factor for persistent postoperative pain [30] may be supported by our study, which shows that the same cohort of patients who had poor surgical outcomes [29] also scored high on catastrophizing. If so, screening the patient's psychological status and preoperative treatment of such disorders may be necessary to avoid persistent postoperative pain.

Our study had several limitations. First, most of the participants in the control group were selected from the Department of Clinical Dentistry at the University of Bergen and were acquainted with the examiner who evaluated them. The acquaintance between the subjects and the examiner may have affected measurements and given rise to bias. A second limitation was that the exclusion criteria did not include other chronic pain disorders and neurological disorders that might have affected the pain perception or medications such as paracetamol, NSAID's, opioids, and antidepressants that might have affected the pain threshold as well as anxiety and depressive symptoms. Since the patients were recruited consecutively by referrals to the National TMD project at Haukeland University Hospital, other chronic pain disorders, neurological disorders, or medications used by the patients were not considered during the recruitment process but were scrutinized during the thorough multidisciplinary investigation by six different specialists. If there was a need of additional specialist investigation, the patients were further referred before the summation of the investigation was presented for the patient. A third major limitation was that there were two different examiners, one examiner for the control group and one for the TMD patient group. The examiners were trained prior to the examination of participants to minimise variabilities and achieve acceptable interexaminer reliability. Nevertheless, there may still have been interexaminer differences that could have affected the results. A fourth limitation was the drop-out of participants in the algometer test due to either no assessments or healthy individuals not wanting to be exposed to the algometer. A final limitation is that the RMS is originally a questionnaire for low-back pain patients, but since it shows disability when living with pain, and because the questions can apply to any type of body pain, it should not affect the validity. An additional shortcoming is that part of the results was published earlier, the psychometric data, but at this time using single independent variables [34]. This can affect the results to be not that relevant or less novel. But after using more advanced statistical analysis which strengthened the results, we found it motivated to let the results be disseminated via a research publication.

## 5. Conclusions

To conclude, the TMD patients have lower pain thresholds and a higher disability of pain compared to healthy individuals. The strongest prediction for TMD was catastrophizing. These results are important to be considered when managing TMD patients. Awareness of psychometric disabilities in TMD patients is of importance and should be addressed in the treatment plan.

## Figures and Tables

**Table 1:** Experimental induced sensitivity or pain thresholds and scores for disability when living with pain and psychometric variables in TMD patients compared to healthy controls.

Measure	Controls		Patients		*P* value
	Mean (SD)	Median	Mean (SD)	Median	
PPT finger	553 (235.6)	516.05	402 (178.1)	375.3	0.001
PPT masseter	246 (106.3)	211.72	168 (81.4)	167.7	0.001
PPT TMJ	225 (112.9)	202.20	157 (69.6)	156.3	0.003
EPT finger	12.94 (6.29)	11.50	11.10 (6.27)	10	0.014
EST finger	4.66 (1.42)	4.00	4.36 (1.15)	4.00	0.185
RMS	0.86 (2.15)	0.00	7.25 (4.11)	7.00	0.001
HADS anxiety*∗*	3.22 (2.98)	2.00	7.12 (4.83)	6.00	0.001
HADS depression*∗*	1.36 (1.99)	1.00	5.83 (4.67)	5.00	0.001
Catastrophizing*∗*	1.33 (2.44)	2.44	7.16 (2.47)	8.00	0.001

Notes: the Wilcoxon signed-rank test was used for group comparison. Abbreviations: PPT = pressure pain threshold; TMJ = temporomandibular joint; EPT = electrical pain threshold; EST = electrical sensibility threshold; RMS = Roland Morris Disability Questionnaire (0–24p); HADS = Hospital Anxiety and Depressions Scale. Units: PPT = kPa; EPT/EST = 0–99; RMS = 0–24p; HADS anxiety = 0–21p; HADS depression = 0–21p; catastrophizing = 0–12p. *∗*These results have been published before by Staniszewski et al. in 2018.

**Table 2:** Unadjusted and adjusted regression analysis of TMD patients.

Independent variables	Unadjusted			Adjusted		
	OR	95% CI for OR	*P* value	OR	95% CI for OR	*P* value
PPT finger (*n* = 45)	0.99	0.99–0.99	0.004	—	—	—
PPT masseter (*n* = 47)	0.10	0.01–0.98	0.001	—	—	—
HADS anxiety (*n* = 60)	1.23	1.10–1.38	0.001	—	—	—
HADS depression (*n* = 59)	1.63	1.27–2.08	0.001	—	—	—
Catastrophizing (*n* = 57)	1.90	1.34–2.72	0.001	2.42	1.22–4.79	0.01

Notes: conditional logistic regression including both unadjusted analysis (Wald test) and adjusted (stepwise forward) analysis with temporomandibular disorders (TMDs) and matched control as dependent variables and with two experimental pain measurements and three self-reported psychometric variables as independent variables. Nagelkerke *R*^2^ = 0.917. Abbreviations: PPT = pressure pain threshold; HADS = Hospital Anxiety and Depression Scale; *n* = number of individuals included in the analysis; SD = standard deviation; OR = odds ratio; units: PPT = kPa; HADS anxiety = 0–21p; HADS depression = 0–21p; catastrophizing = 0–12p.

## Data Availability

The data used to support the findings in the present study are available from the corresponding author upon request.
